# Venous hematology, biochemistry, and blood gas analysis of free-ranging Eastern Copperheads (*Agkistrodon contortrix*) and Eastern Ratsnakes (*Pantherophis alleghaniensis*)

**DOI:** 10.1371/journal.pone.0229102

**Published:** 2020-02-14

**Authors:** Anthony J. Cerreta, Sarah A. Cannizzo, Dustin C. Smith, Larry J. Minter

**Affiliations:** 1 Department of Clinical Sciences, College of Veterinary Medicine, North Carolina State University, Raleigh, North Carolina, United States of America; 2 Fort Worth Zoo, Fort Worth, Texas, United States of America; 3 North Carolina Zoo, Asheboro, North Carolina, United States of America; Copenhagen University Hospital Holbæk, DENMARK

## Abstract

Hematology, plasma biochemistry, and blood gas analysis were performed on venous samples obtained from free-ranging Eastern Copperheads (*Agkistrodon contortrix*) and Eastern Ratsnakes (*Pantherophis alleghaniensis*) in central North Carolina during a mark-recapture study conducted from April to October 2015 at the North Carolina Zoo. Blood samples were collected from 31 (15 male and 16 female) free-ranging copperheads and 34 (20 male and 14 female) free-ranging ratsnakes at the beginning and end of restraint. Restraint was performed for morphometric measurements, sex determination, and identification via placement of intracelomic passive integrated transponder (PIT) tags and marking of ventral scutes with a handheld electrocautery unit. Blood gas analytes were measured at the beginning of restraint and compared to analytes measured at the end to evaluate for changes secondary to handling. Total restraint time prior to the first blood sampling was 1.4 ± 0.4 mins (mean ± SD) and 1.0 ± 0.2 mins (mean ± SD) and restraint time prior to second blood sampling was 12.5 ± 2.4 mins (mean ± SD) and 13.5 ± 3.4 mins (mean ± SD) for copperheads and ratsnakes, respectively. Blood lactate concentrations at the beginning of restraint were similar for both species. Lactate concentrations increased significantly and pH decreased significantly for both species at the end of restraint when compared to the beginning of restraint. Furthermore, lactate concentrations at the end of restraint were significantly elevated in ratsnakes compared to copperheads. This study provides guidelines for interpretation of venous hematology, plasma biochemistry, and blood gas values for free-ranging copperheads and ratsnakes in central North Carolina and demonstrates the physiological response to venous blood gas analytes secondary to capture and restraint.

## Introduction

Eastern Copperheads (*Agkistrodon contortrix*), a venomous snake of the subfamily Crotalinae in the family Viperidae and the nonvenomous colubrid *Pantherophis alleghaniensis*, commonly called the Eastern Ratsnake, are endemic to North America and commonly exhibited in natural history museums, zoological facilities, and aquariums throughout the United States. Despite their popularity in managed collections, published hematologic, biochemical, and blood gas reference values for these species are limited. Baseline physiological data serve as an important reference for the health assessment of both free-ranging and managed populations [1–4]. The increased availability of portable point-of-care analyzers in veterinary medicine has facilitated evaluation of clinical pathology analytes, thus permitting plasma biochemistry and venous blood gas analysis to be conducted in field settings [5,6].

In addition to assessing health status in the field, data from point-of-care analyzers can be used to evaluate the physiological effects of different capture methods on free-ranging animals. Increases in plasma lactate levels have been attributed to stressful events in both humans and avian species [7,8]. This increase in lactate concentration has also been reported in sea turtles following two separate capture techniques and during the manual restraint of free-ranging birds for banding and morphometric data collection [9–11]. Rising lactate concentrations often indicate anaerobic metabolism, thus lactate measurements by portable point-of-care analyzers may provide valuable information regarding decreased tissue perfusion and the physiological effects of exertion or restraint [12].

In this study, hematological, biochemical, and blood gas parameters from 31 copperheads and 34 ratsnakes were evaluated to develop guidelines for the interpretation of these parameters in these species. Venous blood gas analytes were assessed at the beginning and end of restraint to evaluate the impact of capture and restraint on these values. It was hypothesized that lactate concentration, a marker associated with exertion and stress, would significantly increase between the beginning and end of handling due to the stress of restraint in both species and that there would be a concurrent decrease in pH. It was hypothesized that there would be no difference in lactate concentration or pH between the two species.

## Materials and methods

### Ethics statement

This research was conducted within guidelines and approval of the North Carolina Zoo Research Committee. All handling and sampling procedures were consistent with standard vertebrate protocols and veterinary practices, and all efforts were made to minimize pain.

### Animals, sample collection, and sample handling

This study was performed in conjunction with a mark-recapture study conducted on the grounds of the North Carolina Zoo (Asheboro, NC) between 3 April 2015 and 12 October 2015. Peripheral venous blood samples were collected from 31 (15 male and 16 female) free-ranging copperheads and 34 (20 male and 14 female) free-ranging ratsnakes at the beginning and end of restraint. Restraint was performed for morphometric measurements, sex determination, and identification via placement of intracelomic passive integrated transponder (PIT) tags and marking of ventral scutes with a handheld electrocautery unit.

Zoo employees or visitors reported snake sightings, and personnel trained to handle venomous snakes responded to each sighting. All snakes were manually restrained. Non-venomous species were manually restrained by grasping snakes at the base of the head and supporting the body of the snake, as previously described [13]. Venomous species were manually restrained with the head and cranial half of the body in a clear, open-ended, acrylic tube (McMaster-Carr, Douglasville, GA) according to previously described methods [14]. Blood was collected from the ventral coccygeal vein with a 3 mL non-heparinized syringe using a 22-gauge or 25-gauge needle. The first blood sample was collected at the beginning of restraint. Next, cloacal probing was performed to determine the sex of each snake [15]. Morphometric measurements for each snake were then obtained by weighing and measuring total length (TL) and snout-vent length (SVL) using a flexible tape measure. Each snake was given two unique identifiers. Passive integrated transponders (PIT) (Biomark, Boise, ID) were placed in the caudal coelomic cavity and the ventral scutes were marked with a handheld electrocautery unit corresponding to the snake’s identification number [16,17]. After the morphometric measurements, sex determination, and identification procedures were complete, a second blood sample was obtained from the ventral coccygeal vein as previously described. The total blood sample for both collections was less than 1% of the body mass. The total time (mins) from first contact to collection of the first blood sample, and from first blood collection to collection of the second blood sample, were recorded. Based on examinations performed in the field, no abnormalities were detected on physical examination. Snakes were returned to their collection site immediately after the second blood sample was acquired. Individual snakes were only included in this study once.

Hematology, plasma biochemistry, and venous blood gas analysis were performed on the first blood sample (beginning-restraint). The second sample (end-restraint) was used for venous blood gas analysis. Blood smears were made in the field immediately after blood collection from the non-heparinized syringe. The remaining blood was placed into a lithium heparin-coated microtainer tube (BD Microtainer, Becton Dickinson, Franklin Lake, NJ). Any samples that had visible evidence of a blood clot or lymph contamination during venipuncture were excluded.

#### Hematology

Non-heparinized microhematocrit tubes were filled from the lithium heparin-coated microtainer tubes and centrifuged to determine the packed cell volume (PCV). Plasma total solids (TS) were measured via a refractometer (Schuco clinical, Allied Healthcare, St. Louis, MO) and used as a correlate for plasma protein. Blood smears were air dried, heat-fixed, and stained with a modified Giemsa stain (Protocol Hema 3, Fisher Scientific, Kalamazoo, MI). Each slide was first assessed for quality to ensure cells were evenly distributed and that there was no evidence of clumping prior to interpretation. Estimated white blood cell (WBC) counts were measured by taking the average WBC count of 10 fields in the monolayer region viewed at 40X and multiplying by 1700 [18,19]. A minimum of 200 WBCs were counted for each sample to determine the differential leukocyte count. Leukocytes were classified as heterophils, lymphocytes, monocytes, azurophils, eosinophils, or basophils based on cell morphology [18]. The presence of hemogregarine-like parasites, appearing as oblong basophilic intraerythrocytic inclusions in erythrocytes, was classified based on morphology and noted as a comment [20].

#### Plasma biochemistry

Plasma biochemical assays were performed using a tabletop analyzer (VetScan VS2, Avian/Reptilian Profile Plus, Abaxis, Union City, CA). Plasma samples were analyzed for aspartate aminotransferase (AST), creatine kinase (CK), uric acid (UA), glucose (Glucose_pc_), total calcium (Ca), phosphorus (P), total protein (TP), albumin (Alb), globulin (Glo), potassium (K_pc_), and sodium (Na_pc_). Assay methods are displayed in Table 1. Globulin is a calculated value (total protein–albumin). No biochemical analyses were performed on whole blood.

**Table 1:** VetScan assay methods, precision and calibration.

Analytes	Assay	Coefficient of Variation (%)	Calibration Standard	Calibration Method
Aspartate aminotransferase (U/L)	L-aspartate +αketoglutarate	2.0	IFCC	Colorimetric
Creatine kinase (U/L)	Creatine phosphate+ADP	6.0	IFCC	Enzymatic
Uric acid (mg/dL)	Uricase+peroxidase	4.8	Correlation to Beckman LX-20/DX-20	Enzymatic
Glucose (mg/dL)	Hexokinase	1.6	NIST SRM #909	Enzymatic
Calcium (mg/dL)	Arsenazo III method	3.4	Correlation to Beckman LX-20/DX-20	Arsenazo III Dye
Phosphorus (mg/dL)	Sucrose phosphorylase +phosphoglucomutase	4.9	NIST SRM #909B	Enzymatic
Total Protein (g/L)	Biuret reaction	1.9	NIST SRM #909	Biuret (Copper II)
Albumin (g/L)	Bromcresol green dye-binding assay	4.3	Correlation to Beckman LX-20/DX-20	Dye Binding Bromocresol Purple
Potassium (mmol/L)	Pyruvate kinase, lactate dehydrogenase	6.3	NIST SRM #909	Enzymatic
Sodium (mmol/L)	B-galactosidase	1.8	Correlation to Beckman LX-20/DX-20	Enzymatic

Assay methods, coefficients of variation, and calibration standards and methods for biochemistry analytes using the VetScan. When several CVs were reported by the manufacturer, the largest was selected for this study.

IFCC indicates International Federation of Clinical Chemistry; NIST, National Institute of Standards and Technology; SRM, Standard Reference Manual

Repeated analysis on the same blood specimen to assess analyzer between run variability was not performed due to the low specimen volume and cost. Therefore, coefficients of variation (CV) for each analyte were obtained from the manufacturer-reported quality control available on the product inserts (Table 1). The CVs were determined using avian plasma samples for the VetScan Avian/Reptilian Profile Plus rotors. The VetScan uses internal calibrators (in-house calibrators) or reference materials (National Institute of Standards and Technology) to calibrate each parameter. The in-house calibrators are assigned by the previous in-house calibrators, which are considered the gold standard, and verified using a comparative and/or reference method. The methods and materials used in control value assignment procedures are traceable to the standards listed in Table 1.

#### Blood gas analysis

Venous blood gas analyses was performed in the field immediately after collection. A portable point-of-care analyzer (Elemental POC^TM^ Rapid Blood Analyzer, Heska, Loveland, CO) using Element POC^TM^ Test Cards and a subsample of whole blood (0.1 mL) from the lithium heparin tube was used for blood gas analyses. The point-of-care analyzer measured the following analytes: pH, partial pressure of carbon dioxide (pCO_2_), partial pressure of oxygen (pO_2_), sodium (Na), chloride (Cl), potassium (K), ionized calcium (iCa), creatinine, glucose, lactate, hematocrit (HCT), total carbon dioxide (TCO_2_), bicarbonate (HCO_3_^-^), base excess of extracellular fluid (BE(ecf)), base excess of blood (BE(b)), anion gap (AG), oxygen saturation (sO_2_), and hemoglobin (Hgb). The point-of-care analyzer calculated the following analytes: TCO_2_, HCO_3_^-^, BE(ecf), BE(b), AG, sO_2_, and Hgb. The ionized calcium reported was measured at actual pH. The partial pressure of oxygen was not reported because strict anaerobic conditions were not maintained. The following analytes were also not reported: creatinine, sO_2_, BE, Hgb, and HCT. Creatinine is not considered diagnostic in reptiles and oxygen saturation, BE, Hgb, and HCT are measured or calculated using assumptions based on human hemoglobin properties which are not accurate in reptiles [21–23]. Values for pH, pO_2_ and pCO_2_ are presented as uncorrected and corrected based on ambient temperature (T_A_). We performed temperature corrections using the following formulae [24–26].

pH (T_A_) = pH_I_—0.0147 x (T_A_—37) + 0.0065 x (7.4—pH_I_) x (T_A_—37)

pO_2_ (T_A_) = pO_2I_ x 10^−0.0058*(T^_A_^-37)^

pCO_2_ (T_A_) = pCO_2I_ x 10^0.0019*(T^_A_^-37)^

HCO_3_-(T_A_) = αCO_2_ x pCO_2_(T_A_) x 10^(pH(T^_A_^)-pKa)^

TCO_2_(T_A_) = HCO_3_ - (T_A_) + (αCO_2_ x pCO_2_(T_A_))

αCO_2_ = 9.174 x 10^−2^–3.269 x 10^−3^ x T_A_ + 6.364 x 10^−5^ x T_A_^2^–5.378 x 10^−7^ x T_A_^3^

pKa = 6.398–1.341 x 10^−2^ x T_A_ + 2.282 x 10^−4^ x T_A_^2^–1.516 x 10^−6^ x T_A_^3^—log(1.011 + 10^pH(T^_A_^)+0.011xT^_A_^-10.241^ + 10^pH(T^_A_^)+0.001xT^_A_^-8.889^)

#### Statistical analysis

Descriptive statistics including mean, median, standard deviation (SD), and range (i.e. minimum and maximum values) were calculated for all variables. We assessed the data for normality using the Anderson–Darling test (Reference Value Advisor) [27,28]. We then used the Dixon test and the Tukey test to identify outliers and suspect outliers (Reference Value Advisor) [27]. Following the recommendations found in the Guidelines of the American Society for Veterinary Clinical Pathology [29], we calculated reference intervals (90% of reference values) by the robust method after Box–Cox transformation of the data. The number of animals for some analytes assessed in this study do not fully comply with the strict ASVCP guidelines for establishing a reference interval; therefore, these results should only be considered as an estimate rather than true reference ranges [29].

Statistical analysis for morphology (weight, snout-vent length, and total length), hematology, and plasma biochemistry data were analyzed with JMP Pro, Version 12.0.1 (SAS, Cary, NC). Differences by sex for each species were assessed with the Wilcoxon ranked sum. Venous blood gas analytes were compared between the species with the Wilcoxon ranked sum. Blood gas analytes collected at the beginning and end of restraint for each species were compared with the Wilcoxon signed rank test. Paired blood gas analyses from samples obtained at the beginning and end of restraint were available for eight copperheads and 13 ratsnakes. Blood gas results were not analyzed by sex due to small sample size. A chi-squared test was used to compare electrolyte values from the Abaxis and venous blood gas analyzers. The relationships between restraint time and end-restraint lactate concentrations for each species were assessed with Kendall’s τ rank correlation. Statistical significance for all analyses was set at *P* < 0.05.

## Results

Morphometric data for copperheads and ratsnakes are reported in “Table 2”. Male copperheads were significantly heavier and longer than their female counterparts (*P* = 0.02, *P* = 0.03, and *P* = 0.03; weight, snout-to vent length, and total length, respectively), but there was no significant difference in size between male and female ratsnakes (*P* = 0.20, *P* = 0.14, and *P* = 0.18; weight, snout-to vent length, and total length, respectively) (Table 2). Ambient temperature was 26.4°C ± 3.4°C (mean ± SD) and ranged from 20.4°C to 33.9°C throughout the duration of this study.

**Table 2:** Copperhead and ratsnake morphometric parameters.

**A**	**Male (*n* = 14)**				**Female (*n* = 16)**			
**Parameters**	**Mean**	**SD**	**Median**	**Range**	**Mean**	**SD**	**Median**	**Range**
Weight (g)	240*	96.9	255	100–410	134*	44.1	130	40.0–200
Snout-to-vent length (cm)	67.3*	12.5	66.5	49.0–87.0	52.0*	5.50	52.7	36.0–59.0
Total length (cm)	77.5*	13.4	77.8	57.5–99.0	59.8*	6.10	60.5	42.4–67.2
**B**	**Male (*n* = 20)**				**Female (*n* = 14)**			
**Parameters**	**Mean**	**SD**	**Median**	**Range**	**Mean**	**SD**	**Median**	**Range**
Weight (g)	529	383	388	52.0–1500	334	199	322	58.0–700
Snout-to-vent length (cm)	111	28.9	109	61.0–155	98.0	23.4	99.0	64.0–132
Total length (cm)	133	34.0	131	74.0–187	117	27.7	119	77.0–156

Body weight, snout-to-vent length, and total length for male and female free-ranging E. Copperheads (*Agkistrodon contortrix*) (A) and free-ranging E. Ratsnakes (*Pantherophis alleghaniensis*) (B) from central North Carolina. Significant differences between the sexes (*P* < 0.05) are denoted with an asterisk (*).

PCV, TS, estimated WBC counts and differential results are reported in “Table 3” (copperheads) and “Table 4” (ratsnakes). There were no statistically significant sex-specific differences in hematological values for either copperheads (Table 3) or ratsnakes (Table 4). Hemogregarine-like parasites were observed in 20% (12/60) of all snakes captured in this study. The hemoprotozoa genera could not be identified based on morphology [4,20,30,31]. Ten percent of copperheads (3/29) were infected with hemogregarine-like parasites, while 29% (9/31) of ratsnakes were infected.

**Table 3:** Copperhead hematology values.

Analytes	Mean	SD	Median	Min–Max	RIA	LRL 90% CI	URL 90% CI
PCV (%)^a^	28	5.7	29	13–38	12–38	11–20	36–45
Total solids (g/L)^a^	55	10	54	40–74	33–78	31–41	69–85
Est. WBC (10^3^/μL)	13	6.8	11	3.6–27	3.8–32	2.8–5.1	23–39
Heterophils (10^3^/μL)	0.76	0.49	0.67	0.07–2.1	0.08–2.0	0.04–0.18	1.6–2.6
Bands (10^3^/μL)	0	0	0	0	0	0	0
Lymphocytes (10^3^/μL)	8.8	5.7	6.6	1.6–20	1.5–26	1.1–2.1	18–33
Monocytes (10^3^/μL)	0.5	0.9	0.23	0–0.33	0–0.4	0–0.05	0.28–0.49
Eosinophils (10^3^/μL)	0.01	0.01	0	0–0.07	0–0.09	0–0.03	0.04–0.14
Basophils (10^3^/μL)	0.73	0.35	0.71	0.08–1.3	0.04–1.5	0–0.2	1.3–1.7
Azurophils (10^3^/μL)	1.4	1.4	1.3	0–5.7	0–5.4	0–0.9	3.2–5.6

PCV, total solids, and estimated WBC and differential results for free-ranging E. Copperheads (*Agkistrodon contortrix*) from central North Carolina (*n* = 29 unless otherwise noted).

^a^*n* = 17

PCV indicates packed cell volume; WBC, white blood cell; RIA, reference interval approximation; LRL, lower reference limit; URL, upper reference limit

**Table 4:** Ratsnake hematology values.

Analytes	Mean	SD	Median	Min–Max	RIA	LRL 90% CI	URL 90% CI
PCV (%)^a^	27	5.4	28	14–38	14–37	7.0–19	35–39
Total solids (g/L)^a^	62	13	61	40–92	36–91	30–43	81–99
Est. WBC (10^3^/μL)	8.9	3.5	8.7	2.9–16	2.4–17	1.4–3.6	14–19
Heterophils (10^3^/μL)	1.1	0.7	1.1	0.1–3.0	0–2.9	0–0.2	2.4–3.5
Bands (10^3^/μL)	0	0	0	0	0	0	0
Lymphocytes (10^3^/μL)	6.1	3.3	5.6	1.1–14	1.2–15	0.8–1.8	12–17
Monocytes (10^3^/μL)	0.6	0.7	0.4	0–2.0	0–2.2	0–0.8	1.6–2.5
Eosinophils (10^3^/μL)	0.03	0.17	0	0–0.9	0–0.7	0–0.01	0.02–0.8
Basophils (10^3^/μL)	0.19	0.19	0.11	0–0.64	0–0.8	0–0.1	0.5–1.0
Azurophils (10^3^/μL)	0.01	0.01	0	0–0.4	0–0.5	0–0.01	0.2–0.6

PCV, total solids, and estimated WBC and differential results for free-ranging E. Ratsnakes (*Pantherophis alleghaniensis*) from central North Carolina (*n* = 31 unless otherwise noted).

^a^*n* = 22

PCV indicates packed cell volume; WBC, white blood cell; RIA, reference interval approximation; LRL, lower reference limit; URL, upper reference limit

Plasma biochemistry results obtained from the Abaxis are reported in “Table 5” (copperheads) and “Table 6” (ratsnakes). Male copperheads had significantly higher albumin than female copperheads (*P* = 0.04). They also had a significantly higher CK than female copperheads (*P* = 0.04). There were no additional statistically significant sex-specific differences in biochemistry values for either copperheads (Table 5) or ratsnakes (Table 6).

**Table 5:** Copperhead biochemistry parameters.

Analytes	Mean	SD	Median	Range
Aspartate aminotransferase (U/L)	48	85	25	11–342
Creatine kinase (U/L)	837	1170	507	205–4800
Uric acid (mg/dL)	5.0	4.0	2.8	1.5–14.6
Glucose (mg/dL)	51	29	47	19–122
Calcium (mg/dL)	15.1	2.6	14.0	12.0–20.1
Phosphorus (mg/dL)	4.1	1.1	4.1	2.1–6.7
Total Protein (g/L)	52	5.8	52	43–66
Albumin (g/L)	11	1.8	11	7–14
Globulin (g/L) ^a^	42	4.7	41	34–53
Potassium (mmol/L)	6.4	0.5	5.9	4.8–9.8
Sodium (mmol/L)	158	8	157	143–172

Plasma biochemistry values for free-ranging E. Copperheads (*Agkistrodon contortrix*) from central North Carolina (*n* = 14 unless otherwise noted).

^a^*n* = 12

**Table 6:** Ratsnake biochemistry parameters.

Analytes	Mean	SD	Median	Range
Aspartate aminotransferase (U/L)	22	16	18	9–72
Creatine kinase (U/L)	616	615	392	88–2701
Uric acid (mg/dL)	5	6.3	3.0	1.0–25.1
Glucose (mg/dL)	64	14	64	42–95
Calcium (mg/dL)	15.4	1.1	15.3	13.8–18
Phosphorus (mg/dL)	5.1	1.9	4.4	2.5–9.2
Total Protein (g/L)	58	8.3	59	43–74
Albumin (g/L)	21	3	21	14–26
Globulin (g/L)	37	6.2	38	29–49
Potassium (mmol/L)	5.7	1.3	5.7	2.2–7.6
Sodium (mmol/L)	164	7	162	156–180

Plasma biochemistry values for free-ranging E. Ratsnakes (*Pantherophis alleghaniensis*) from central North Carolina (*n* = 18).

Venous blood gas analysis results from the beginning and end of restraint are reported in “Table 7” (not temperature corrected) and “Table 8” (temperature corrected). There were no significant differences in the analytes (sodium, potassium, and glucose) obtained from the Abaxis compared with the venous blood gas analysis (*P* = 0.37, *P* = 0.25 and *P* = 0.41, respectively). The total time (mins) from first contact to collection of the first blood sample, was 1.4 ± 0.4 mins (mean ± SD) and 1.0 ± 0.2 mins (mean ± SD) for copperheads and ratsnakes, respectively. The total time (mins) from first blood collection to collection of the second blood sample, was 12.5 ± 2.4 mins (mean ± SD) and 13.5 ± 3.4 mins (mean ± SD) for copperheads and ratsnakes, respectively. There were no significant differences in restraint times between the two species (*P* = 0.43 and *P* = 0.52, respectively). Lactate concentrations increased significantly between beginning-restraint sample and end-restraint sample in both species (*P* = 0.03 and *P* = 0.01 for copperheads and ratsnakes, respectively). Venous blood pH significantly decreased between beginning-restraint sample and end-restraint sample in both species (*P* = 0.04 and *P* = 0.04 for copperheads and ratsnakes, respectively). End-restraint lactate concentrations were significantly greater in ratsnakes compared to the copperheads (*P* = 0.01).

**Table 7:** Venous blood gas results (not temperature corrected).

	**E. Copperheads**							
	**Beginning-restraint (*n* = 14)**				**End-restraint (*n* = 12)**			
**Analytes**	**Mean**	**SD**	**Median**	**Range**	**Mean**	**SD**	**Median**	**Range**
pH	7.19*	0.21	7.16	6.91–7.62	7.12*	0.22	7.06	6.82–7.45
pCO_2_ (mmHg)	25.3	10.6	21.8	11.8–41.9	27.6	13.6	25.7	7.8–51.6
Sodium (mmol/L)	158	6	157	149–176	158	4	159	150–163
Chloride (mmol/L)	127	7	129	109–138	128	7	130	114–135
Potassium (mmol/L)	5.2	1.1	5.0	3.9–8.0	5.0	0.6	4.9	4.1–5.9
Ionized Calcium (mmol/L)	1.71	0.23	1.71	1.08–2.12	1.71	0.21	1.75	1.16–1.92
Glucose (mmol/L)	2.38	1.55	2.11	1.11–6.94	2.22	0.78	2.33	1.11–3.28
Glucose (mg/dL)	43	28	38	20–125	40	14	42	20–59
Lactate (mmol/L)	10.9*	6.75	9.61	2.13–20.1	12.7*†	5.58	11.6	5.48–20.1
TCO_2_ (mmol/L)	10.4	3.9	10.0	5.0–17.3	9.9	5.1	9.2	3.3–18.5
HCO_3_^-^ (mmol/L)	9.6	3.8	9.1	4.4–16.8	9.0	4.9	8.1	3.1–17.2
Anion Gap (mmol/L)	27	6	25	20–42	27†	4	27†	20–32
	**E. Ratsnakes**							
	**Beginning-restraint (*n* = 19)**				**End-restraint (*n* = 18)**			
**Analytes**	**Mean**	**SD**	**Median**	**Range**	**Mean**	**SD**	**Median**	**Range**
pH	7.30*	0.19	7.33	6.97–7.74	7.22*	0.23	7.18	6.86–7.81
pCO_2_ (mmHg)	36.1	19.2	31.9	11.6–78.5	26.0	10.2	25.3	9.8–45.1
Sodium (mmol/L)	162	5	162	153–180	165	7	164	151–180
Chloride (mmol/L)^a^	124	10	126	93–140	127	9	127	99–140
Potassium (mmol/L)^b^	5.2	1.0	5.1	3.8–8.0	5.5	0.6	5.4	4.6–7.0
Ionized Calcium (mmol/L)^c^	1.51	0.19	1.56	1.17–1.76	1.53	0.22	1.59	0.96–1.87
Glucose (mmol/L)	3.05	0.72	3.22	1.67–4.22	3.72	0.61	3.66	2.39–4.88
Glucose (mg/dL)	55	13	58	30–76	67	11	66	43–88
Lactate (mmol/L)	10.3*	5.37	10.1	2.66–20.1	17.4*†	2.93	17.86	10.98–20.1
TCO_2_ (mmol/L)	17.8	8.2	16.3	8.9–47.5	11.2	3.8	11.6	6.8–18.8
HCO_3_^-^ (mmol/L)	16.7	7.8	15.0	8.6–45.3	10.5	3.8	10.6	6.0–18.5
Anion Gap (mmol/L)^d^	25	5	25	16–34	33	6	32	24–48

First blood sample (beginning-restraint) and second blood sample (end-restraint) blood gas values for free-ranging E. Copperheads (*Agkistrodon contortrix*) and E. Ratsnakes (*Pantherophis alleghaniensis*) from central North Carolina. Significant differences between the sampling periods (*P* < 0.05) for each species are denoted with an asterisk (*); only the paired samples (*n* = 8 for E. Copperheads and *n* = 13 for E. Ratsnakes) were compared. Significant differences between the species (*P* < 0.05) are denoted with a cross (†).

^a^Beginning-restraint *n* = 18

^b^End-restraint *n* = 17

^c^End-restraint *n* = 16

^d^Beginning-restraint *n* = 17, End-restraint *n* = 17

**Table 8:** Venous blood gas results (temperature corrected).

	**E. Copperheads**							
	**Beginning-restraint (*n* = 14)**				**End-restraint (*n* = 12)**			
**Analytes**	**Mean**	**SD**	**Median**	**Range**	**Mean**	**SD**	**Median**	**Range**
pH	7.33*	0.24	7.34	6.99–7.78	7.24*	0.25	7.18	6.91–7.64
pCO_2_ (mmHg)	24.3	10.3	20.6	11.2–40.4	26.3	13.0	24.5	7.4–49.5
TCO_2_ (mmol/L)	9.0	8.4	6.4	2.2–30.4	7.4	6.5	4.4	1.5–20.3
HCO_3_^-^ (mmol/L)	8.1	8.5	5.4	1.6–29.8	6.3	6.6	3.2	0.88–19.5
	**E. Ratsnakes**							
	**Beginning-restraint (*n* = 19)**				**End-restraint (*n* = 18)**			
**Analytes**	**Mean**	**SD**	**Median**	**Range**	**Mean**	**SD**	**Median**	**Range**
pH	7.45*	0.21	7.46	7.07–7.96	7.36*	0.26	7.32	6.93–8.07
pCO_2_ (mmHg)	34.5	18.4	30.0	10.9–75.9	24.9	9.8	24.6	9.4–43.2
TCO_2_ (mmol/L)	18.2	17.2	12.8	7.5–76.4	12.5	19.6	5.9	2.4–86.8
HCO_3_^-^ (mmol/L)	16.9	17	12.2	5.0–73.4	11.6	19.7	5.2	1.2–86.4

First blood sample (beginning-restraint) and second blood sample (end-restraint) blood gas values corrected for ambient temperature for free-ranging E. Copperheads (*Agkistrodon contortrix*) and E. Ratsnakes (*Pantherophis alleghaniensis*) from central North Carolina. Significant differences between the sampling periods (*P* < 0.05) for each species are denoted with an asterisk (*); only the paired samples (*n* = 8 for E. Copperheads and *n* = 13 for E. Ratsnakes) were compared. Significant differences between the species (*P* < 0.05) are denoted with a cross (†).

## Discussion

This study aimed to establish guidelines for the interpretation of venous hematology, plasma biochemistry, and blood gas analytes in free-ranging copperheads and ratsnakes while also assessing for changes in venous blood gas analytes secondary to restraint. This study documented that blood lactate concentrations at the beginning of restraint were similar for both species. Additionally, lactate concentrations increased significantly in both species at the end of restraint when compared to the beginning of restraint. The significant decrease in venous blood pH between the beginning-restraint sample and end-restraint sample in both species likely occurred secondary to the aforementioned changes in lactate.

Although previous studies have reported baseline biochemical, blood gas, and hematology values for other viperid and colubrid snakes, this is the first study to establish guidelines for the interpretation of these analytes in copperheads and ratsnakes. The hematological and plasma biochemical results for copperheads and ratsnakes reported in this study were consistent with previously published values for both viperid and colubrid snakes, with the exception of higher leukocyte counts in the copperheads [4,32–39]. This difference may be attributed to many variables such as species, age, size, time of year, methodology, and instrumentation [4,34,40]. Due to the nucleated nature of reptile erythrocytes, automated methods of obtaining white blood cell (WBC) counts and differentials are not accurate; manual methods are utilized in these species, with the accuracy of results dependent on the cytologist’s experience [18]. In this study, estimated leukocyte counts and differentials were performed with the standard protocol used at the North Carolina Zoo, where the monolayer regions of the blood smear are scanned for estimating the total number of leukocytes.

Point-of-care analyzers are valuable diagnostic tools, especially in field settings or medical emergencies where rapid results are needed. Commonly used point-of-care analyzers perform blood gas analysis that assess respiratory function, metabolic status, and tissue perfusion, and provide near-instantaneous results [41–43]. However, many of the blood gas components are influenced by temperature, so when applied to research involving reptiles and other poikilotherms, clinicians and researchers must decide whether to correct the results for ambient temperature. Temperature corrections may not always be warranted and instituting them can preclude comparison of data sets if the methods are not stated, vary among sources, or if the temperatures are different [44–46]. There are multiple formulae for manually performing temperature corrections, but none have been validated for snake blood gas analysis performed with portable point-of-care analyzers, such as the iSTAT (Abbott Point of Care, Princeton, NJ) or the EPOC [9,22,47,48]. Venous blood gas results from copperheads and ratsnakes are presented both without temperature correction “Table 7” and with temperature correction to ambient temperature “Table 8” to facilitate comparison with future studies and clinical applications. Temperature corrections did not affect statistical comparisons between copperheads and ratsnakes.

Despite all of the snakes in this study appearing outwardly healthy on physical examination, lactate concentrations both at the beginning and end of restraint were higher than the reported lactate concentrations from other reptiles, except cold-stunned Kemp’s ridley sea turtles on their first day of rehabilitation and manually restrained Nile crocodiles [5,43,47–53]. The higher lactate concentrations in copperhead and ratsnakes may reflect species-specific differences in anaerobic metabolism or differences in instrumentation, however, additional considerations for elevated lactate in reptiles include hepatocellular damage, renal disease, hypoventilation, poor perfusion, and cardiovascular disease [12,54]. Based on physical examinations, there was no evidence for these conditions in the animals used in this study. These comparisons illustrate the need for species-specific reference intervals and underscore the importance of understanding clinicopathologic responses to restraint. Improved understanding of the effects of capture and restraint in these species will enable clinicians to interpret lactate results more appropriately and improve clinical reasoning.

The lactate concentrations from the samples obtained at the beginning and end of restraint for copperheads were comparable to lactate concentrations observed in male copperheads 60 minutes after losing intra-sexual conspecific fights [55]. Both the winners of these ritualized competitions and control animals, which did not fight, were found to have lower lactate concentrations than the copperheads in this study; however, these animals were anesthetized prior to blood collection which may have reduced their level of stress and lactate production [55]. Due to the similarity of lactate concentrations from our study to those in studies on the metabolic costs of reproductive behaviors [55], the restraint of animals may be as physiologically demanding as the physical interactions that occur during the breeding season.

Blood gas analyses, and more specifically lactate measurements, can serve as a means of evaluating the physiological response to capture, restraint, and human interactions in snakes; however, the physiological response to restraint techniques and handling can vary by species [10,56]. In this study, lactate concentrations increased and pH decreased in both species when the first blood sample (beginning-restraint) and second blood sample (end-restraint) were compared, but lactate was significantly increased in ratsnakes at the end of restraint. The significant increase in ratsnakes may be associated with a stronger sympathetic nervous system response to the more hands-on restraint that these non-venomous snakes experienced. This increase in sympathetic nervous system response may have resulted in increased physical resistance to restraint and subsequently, higher end-restraint lactate concentrations. In contrast, copperheads were restrained in open-ended, acrylic tubes, which reduced the amount of hands-on contact. Our results differ from Kreger and Mench [56] who found higher corticosterone concentrations in Ball Pythons (*Python regius*) restrained in polyvinyl chloride (PVC) restraint tubes compared to non-handled controls while animals that underwent handheld restraint did not exhibit significantly different corticosterone concentrations compared to controls. It is possible that lactate and corticosterone are not interchangeable proxies for measuring response to restraint. Alternatively, differences in resting metabolic rate between pit vipers and colubrids may explain the differences in lactate following restraint. These colubrids are active foragers and generally have higher resting metabolic rates than many pit vipers which are ambush foragers [57,58]. Thus, the higher resting oxygen requirements of ratsnakes could translate into an earlier switch to anaerobic metabolism during restraint, which would be reflected in higher post-restraint lactate.

A shift from aerobic to anaerobic metabolism often results from failure of the oxygen supply system in meeting the energy demands of tissues. In reptiles, this shift occurs to varying degrees in response to high energy utilization during intense muscular activity, or anoxia in aquatic species [59,60]. Increased lactate concentrations can lead to acidemia, which potentiates the anaerobic cycle and may lead to electrolyte imbalances [61]. Point-of-care analyzers enable clinicians to perform analyses to elucidate the metabolic status of a patient in the field, without the delay imposed by measuring these parameters in a laboratory. The physiologic effect of handling on snakes may be apparent when the pH and lactate results of this study are compared to results from South American Rattlesnakes (*Crotalus durissus terrificus*), which had arterial catheters with long extensions to eliminate the effects on handling on lactate results [52]. The lower pH and higher lactate results in copperheads and ratsnakes compared to the non-handled rattlesnakes suggests that even brief handling has an immediate effect on metabolism and subsequently, blood pH and lactate. The role of species differences in response to restraint and metabolism as well as differences in the instrumentation used to assess these parameters may also affect these results; thus further research is necessary to establish reference intervals to facilitate interspecies comparisons.

The pCO_2_ and HCO_3_^-^ results in this study were lower than those reported in other reptiles including Marine Iguanas (*Amblyrhynchus cristatus*), anesthetized Green Iguanas (*Iguana iguana)*, Inland Bearded Dragons (*Pogona vitticeps*), and sea turtles [5,43,47,48,50,53,62–64]. In conjunction with the metabolic lactic acidosis detected in the snakes of this study, the observed decrease in pCO_2_ and HCO_3_^-^ concentrations were interpreted as a correction by hyperventilation (compensatory respiratory alkalosis) and the titration of HCO_3_^-^ by circulating lactic acid. The lower pCO_2_ and HCO_3_^-^ concentrations in both snake species could also represent taxonomic differences. Lower HCO_3_^-^ may also be explained by air exposure during sample processing, but Kirshbaum [65] found minimal changes in HCO_3_^-^ concentrations in serum samples exposed to air for up to two hours.

This study provides baseline venous hematology, plasma biochemistry, and blood gas data for free-ranging copperheads and ratsnakes. These results add to a growing database of knowledge about health management in wild and managed reptiles. Although the sample size for some analytes in this study do not meet the specifications for formal determination of reference intervals [28], these results provide researchers and veterinarians with species-specific guidelines, which will be valuable for field health assessments and captive management of these species. Future research is necessary to establish formal reference intervals for these species to facilitate comparisons of blood values across age groups, geographical localities, and subspecies.

This study also demonstrates free-ranging copperheads and ratsnakes’ physiological response to capture and restraint. These differences should be considered when interpreting the results of blood chemistry analysis and when planning medical procedures and population studies in the field. Further research comparing the two methods of restraint used in this study may contribute to our understanding of this physiological response and improve animal welfare in scenarios necessitating restraint. An improved understanding of the effects of restraint in reptiles will enable researchers and clinicians to interpret venous blood gas results more appropriately.

## Conclusions

- This study provides guidelines for the interpretation of venous hematology, plasma biochemistry, and blood gas values for free-ranging copperheads and ratsnakes in central North Carolina.
- Capture and restraint resulted in a significant increase in lactate and a significant decrease in pH for both species, underscoring the effects of stress and handling on clinicopathologic data.

## Supporting information

S1 File (XLSX)
